# Comparison of the effect of oral and vaginal misoprostol on labor induction: updating a systematic review and meta-analysis of interventional studies

**DOI:** 10.1186/s40001-023-01007-8

**Published:** 2023-01-27

**Authors:** Maryam Rahimi, Ladan Haghighi, Hamid Reza Baradaran, Mobin Azami, Samaneh Saghafian Larijani, Paniz Kazemzadeh, Yousef Moradi

**Affiliations:** 1 Department of Gynecology and Obstetrics, School of Medicine, Iran University of Medical Sciences, Tehran, Iran; 2 Ageing Clinical & Experimental Research Team, Institute of Applied Health Sciences, University of Aberdeen, Aberdeen, UK; 3 Department of Epidemiology, School of Public Health, Iran University of Medical Sciences, Tehran, Iran; 4 Student Research Committee, Kurdistan University of Medical Sciences, Sanandaj, Iran; 5 Social Determinant of the Health Research Center, Research Institute for Health Development, Kurdistan University of Medical Sciences, Sanandaj, Iran; 6 Department of Epidemiology and Biostatistics, School of Medicine, Kurdistan University of Medical Sciences, Sanandaj, Iran

**Keywords:** Oral misoprostol, Vaginal misoprostol, Labor induction, Systematic review, Meta-analysis

## Abstract

**Objectives:**

This study is aimed to compare the effect of oral misoprostol with vaginal misoprostol to induce labor as a systematic review and meta-analysis.

**Methods:**

Electronic databases including PubMed [Medline], Scopus, Web of science, Embase, Ovid, Cochrane library, and ClinicalTrials.gov were searched using the relevant keywords. All RCTs comparing the effect of oral vs vaginal misoprostol on labor induction were considered. The Cochrane Risk of Bias checklist was used for assessing quality of included RCTs. All statistical analyses were completed using STATA (Version 16) and Revman (Version 5).

**Results:**

Thirty-three RCTs with 5162 patients (1560 in oral and 2602 in vaginal groups) were included in this meta-analysis. Labor induction length did differ significantly between the two routes of misoprostol administration [Standardized Mean Difference: 0.40 h, 95% confidence interval (CI) 0.34, 0.46; I^2^: 66.35%; *P* = 0.04]. In addition, the risk of neonatal death, tachysystole, uterine hyperstimulation, preeclampsia, non-FHR and abortion was lower in the oral misoprostol group and the risk of hypertonus, PROM, oxytocin need and cesarean fever was higher in this group than the vaginal misoprostol group.

**Conclusions:**

Based on results of this meta-analysis, it can be inferred that currently, clinical specialists can decide to use this drug orally or vaginally on a case-by-case basis, depending on the condition of the pregnant mother and the baby.

**Supplementary Information:**

The online version contains supplementary material available at 10.1186/s40001-023-01007-8.

## Background

Induction of labor means stimulation of uterine contractions before the onset of spontaneous labor [1, 2] and is indicated in cases where the benefits to the mother or fetus outweigh the ones of continued pregnancy [2]. Factors affecting the success of labor induction can be summarized in multiple pregnancies, body mass index less than 30 kg/M^2^, birth weight less than 3500 gr and favorable cervical conditions [2–4]. One of the few methods to predict the outcomes of labor induction is the bishop scoring system in which factors including cervical dilatation, cervical effacement, presentation organ position, cervical consistency and cervical conditions are used to score. The cervix readiness is important for successful induction of labor [5–7]. The methods used to prepare the cervix include pharmaceutical products and various forms of mechanical cervical dilators. Pharmacological techniques mainly involve the use of prostaglandin products. In the past, the role of the laminaria and E-series of prostaglandins has been proven in cervical dilatation and reduction in its dilatation complications during surgery. Misoprostol is a synthetic analogue of prostaglandin E1, used in the treatment and prevention of gastric ulcers and is widely used today in gynecology and obstetrics [8–10]. Its applications in gynecology and obstetrics include medical abortion in the first and second trimesters of pregnancy, preparation of the cervix before dilatation and evacuation or dilatation and curettage as well as prevention and treatment of postpartum hemorrhage [9–12]. The advantage of misoprostol over other prostaglandin analogues is that it is cheaper, stable at the room temperature and also available in the form of oral tablets. Although misoprostol has been formulated for oral administration, numerous pharmacokinetic studies have shown the concentration of its active metabolite remains in the vaginal administration for a longer time period [9, 13]. For example, in a study by Cem Batukan et al., which examined the effect of vaginal and oral misoprostol on cervical preparation, the results showed vaginal misoprostol prescription was preferable to oral administration [14, 15]. Waleed E Khayat et al. also compared the effect of vaginal isosorbide mononitrate with vaginal misoprostol in cervical preparation and concluded the rate of primary cervical dilatation and the duration of dilatation were higher in the misoprostol group but there was not a statistically significant difference between the two groups in the duration of surgery or difficult dilatation [14, 15]. Based on the results of these studies, vaginal misoprostol is expected to be more effective than oral preparations of the cervix, but clinical trial studies have reported conflicting results [5, 7, 8, 10, 12, 14–17]. Therefore, this study aimed to compare the effect of oral misoprostol with vaginal misoprostol to induce labor as a systematic review and meta-analysis.

## Methods

This article was written based on the Standards of Preferred Reporting Items for Systematic Reviews and Meta-Analyses (PRISMA) [18].

### Search strategy and screening

Intervention studies published from January 1990 to January 2022 in 5 electronic databases (PubMed [Medline], Scopus, Web of science, Embase, Ovid, Cochrane library, and ClinicalTrials.gov) were reviewed using keywords “Misoprostol”, “Induction of Labor”, and “Induced Labor”.

In each electronic database, related keywords were selected using MeSH and EMTREE. Reporting checklist for search strategy was based on PRISMA. The selection criteria were based on the PICO structure, so that the desired Population was pregnant women, Intervention oral use of misoprostol, Comparison vaginal misoprostol and Outcomes were labor induction, drug side effects as well as maternal and neonatal outcomes. Finally, the studies included randomized, cross-sectional, or parallel clinical trial ones. Non-English language studies as well as cohort studies, case studies, clinical trials, letters to the editor and systematic reviews were excluded from this meta-analysis. In addition, articles whose statistical population was other than pregnant women or examined other interventions were removed. To find gray literature, manual search was performed using references of related articles. The search strategy was developed by two independent authors (YM and PK) and the disputes were resolved with the opinion of a third researcher (LH) with more experience.

In the next step, an Endnote (Version 8) library was created to collect articles, remove duplicates, and review titles and abstracts. Initially, the review of titles and abstracts was independently done by the researcher (PK) and 10% of the reviewed articles were randomly reviewed by the second researcher (YM) and disputes were resolved by discussion and referral to the third party (LH) if necessary. The screened references were selected for full-text review if they contained the desired information in the title or abstract. Full text review was separately performed by the two authors (YM and MS). Data were extracted from eligible studies and were entered into Excel 2019.

### Extraction of data

To extract data from the articles, first a checklist was prepared with the opinion of the research team and required information including author name, year of article publication, sample size of study groups, country of the study, age of mothers, dose of the drug in the vaginal and oral misoprostol groups, follow-up period in the study, maternal outcomes (preeclampsia, oligohydramnios, abortion, cesarean section, mean labor duration, uterine tachysystole, uterine muscle strength or traction and oxytocin requirement), drug side effects (nausea and vomiting, headache and fever) and neonatal complications (meconium excretion, the Apgar score less than 7 at the first and fifth minutes, neonatal death, hospitalization in the neonatal intensive care unit (NICU), IUGR and PROM) was extracted.

### Risk of bias

The risk of bias in the included studies was assessed using the Cochrane bias risk tool for interventional studies. Areas of evaluated bias included sequence generation, allocation concealment, blinding, outcome data and outcome reporting. Clinical trial studies, after evaluation by this tool, were classified into low, high, and unclear groups. The two authors independently evaluated the quality of the articles using this tool.

### Statistical analysis

In this meta-analysis, the effect sizes were equal to the standardized mean difference (SMD) and risk ratio. The means reported in the two groups of studies included in the meta-analysis were combined using the DerSimonian–Liar random-effects model and finally the weighted average was reported. To report the risk ratio, the frequency of the desired outcomes in the two intervention and comparison groups was extracted and using the constant effects model, the logarithm and logarithm standard deviation of the risk ratios were combined and finally the pooled risk ratio was reported. Cochrane Q and I2 tests were used to investigate the heterogeneity and variance between the studies selected for meta-analysis. Funnel Plot and Egger test were used to evaluate the publication bias. In addition, the meta-regression analysis and diagram were applied to investigate the association between variables of women's age, the sample size of selected studies and the estimated pooled risk ratio. All two-way statistical tests were considered with *α* = 0.05 and statistical analyses were performed in STATA software version 16 and Revman version 5.

## Results

### Qualitative results

**Fig. 1:**
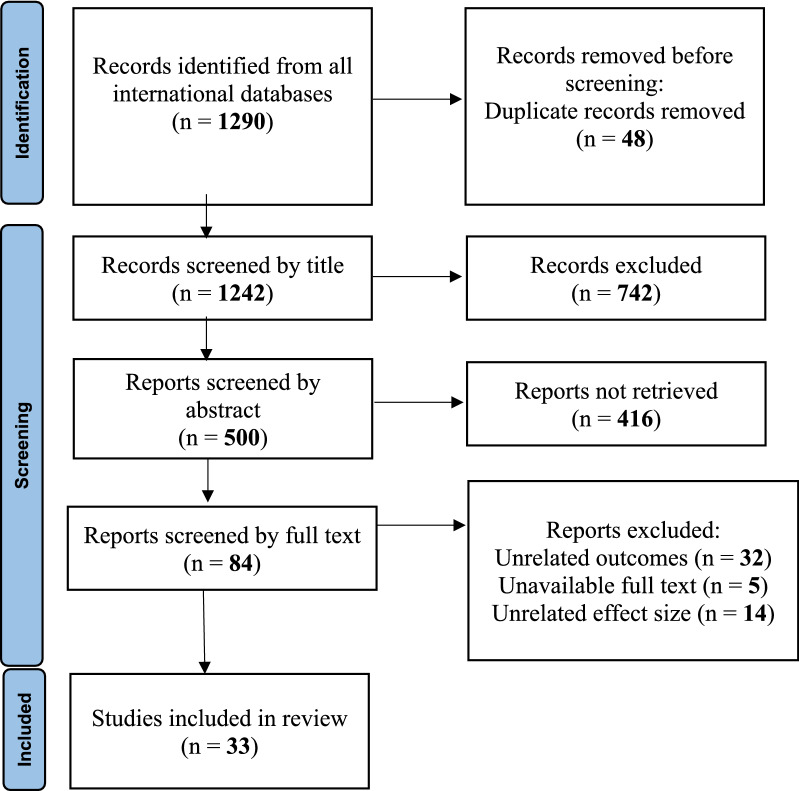
Flow diagram for related article numbers which included in meta-analysis

In this study, after completing the search strategy and searching in international databases, 1290 articles were found. After removing duplicates and screening based on article titles, 500 studies remained, which were entered into the screening phase based on their abstracts. In the next stage, 416 articles were removed due to the irrelevance of their abstracts and type of study with the objectives of the present research and 84 articles were included in the full-text review. After review and evaluation of the full-text of articles, 33 clinical trial studies were entered into the meta-analysis (Fig. 1).

In these 33 clinical trial studies, in 25 studies in the intervention group, pregnant women were orally given 50 µg, in 5 studies 25 µg, in 2 studies 20 µg and in 1 study 100 µg of misoprostol. In the comparison group, where misoprostol was vaginally given, in 17 studies, 25 and in the remaining studies, 50 mg of misoprostol were given to pregnant women. The sample size in all these clinical trial studies was equal to 5362 pregnant women, of whom 2660 were in the oral misoprostol group and 2702 were in the vaginal misoprostol group. The majority of clinical trial studies included in the meta-analysis measured the desired outcomes 4 h after the intervention (Table 1).

**Table 1:** Characteristics of included randomized control trial studies

Authors	Years	Country	Dose oral indevotional	Dose vaginal interventional	Hours of intervention	Total sample	Sample oral indevotional	Sample Vaginal Interventional	Age
Cheng et al.	2008	China	25 mcg	25 mcg	4	207	101	106	28.3
Rezaie et al.	2016	Iran	100 ug	25 ug	4	120	60	60	27.7
Souza et al.	2013	Brazil	20 ug	25 ug	6	200	100	100	23
Sarella et al.	2021	India	20 ug	25 ug	2	80	40	40	22.5
Kaur et al.	2015	India	25 ug	25 ug	4	100	50	50	25.6
Mehta et al.	2020	India	25 ug	25 ug	4	100	50	50	26
How et al.	2001	USA	25 ug	25 ug	4	219	109	110	23.4
Bagariya et al.	2020	India	25 ug	25 ug	4	196	98	98	NR
Rezaie et al.	2016	Iran	50 ug	25 ug	4	120	60	60	28.4
Wing et al.	1999	USA	50 ug	25 ug	4	220	110	110	NR
Komala et al.	2013	India	50 ug	25 ug	4 to 6	200	100	100	NR
Rahman et al.	2013	India	50 ug	25 ug	4	220	110	110	27.2
Colon et al.	2005	USA	50 ug	25 ug	4	204	93	111	28.1
Zvandasara et al.	1999	Zimbabwe	50 ug	25 ug	1	134	69	65	23
Paisarntantiwong et al.	2005	Thailand	50 ug	25 ug	NR	146	73	73	25.6
Galidevara et al.	2018	India	50 ug	25 ug	4	163	80	83	24.4
Sheela et al.	2007	India	50 ug	25 ug	6	100	50	50	24
Deshmukh et al.	2013	India	50 mcg	50 mcg	6	200	100	100	26.4
Nopdonrattakoon et al.	2003	Thailand	50 mg	50 mg	4	106	53	53	24.9
Sheikher et al.	2009	India	50 ug	50 ug	4	60	30	30	NR
Roux et al.	2002	South Africa	50 ug	50 ug	6	240	120	120	28.1
Kwon et al.	2001	Canada	50 ug	50 ug	6	160	78	82	27.2
Bennett et al.	1998	Canada	50 ug	50 ug	4	206	104	102	28.7
Shetty et al.	2001	UK	50 ug	50 ug	4	245	122	123	28
Promila et al.	2011	India	50 ug	50 ug	4	103	51	52	26
Elhassan et al.	2007	Sudan	50 ug	50 ug	NR	100	50	50	NR
Ezechukwu et al.	2015	Nigeria	50 ug	50 ug	24	140	70	70	27.2
Fisher et al.	2001	Canada	50 ug	50 ug	6	126	62	64	27
Young et al.	2020	Canada	50 ug	50 ug	4	339	167	172	29.1
Ambika et al.	2017	India	50 ug	50 ug	6	200	100	100	20
Mehrotra et al.	2010	India	50 ug	50 ug	4	128	60	68	26.24
Adam et al.	2005	Sudan	50 ug	50 ug	6	80	40	40	NR

### Quantitative results

**Fig. 2:**
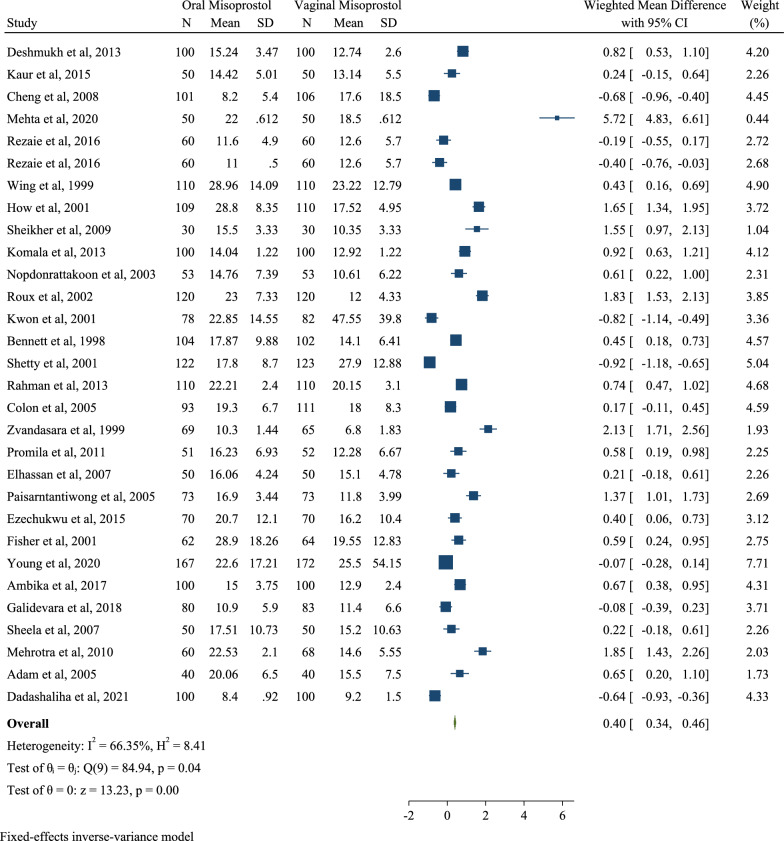
Forest plot for pooled weighted mean difference of induction labor length in oral and vaginal misoprostol groups

The first outcome of this study was calculation of the mean duration of labor, measured in clinical trial studies included in the meta-analysis as the mean and standard deviation. Of the 33 clinical trial studies included in the meta-analysis, 30 reported the mean duration of labor in the both groups. After combining the results of these studies, the difference in the weighted mean was 0.40 with a confidence interval of 0.34 to 0.46 h (SMD 0.40; 95% CI 0.34, 0.46; I2: 66.35%; P: 0.04). Therefore, orally taking misoprostol can be said to increase the duration of labor by 0.40 h compared to vaginally taking this drug (Fig. 2).

**Fig. 3:**
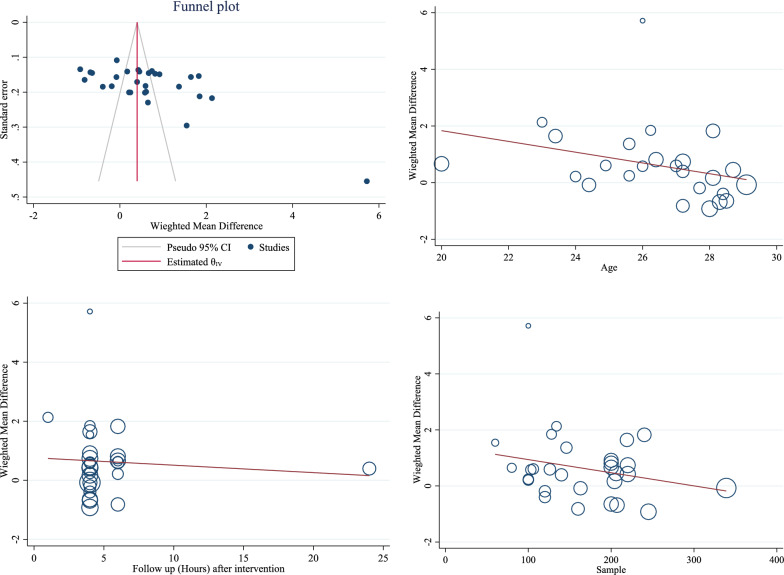
Results of publication bias and effect of age and follow-up duration on pooled weighted mean difference (WMD)

The results of the publication bias showed the publication bias occurred in these clinical trial studies included in the meta-analysis (*B* = 14.67; SE = 2.50; *P* = 0.0001) (Fig. 3). The results of meta-regression also showed the age of pregnant mothers was indirectly related to the duration of labor based on hours but was not statistically significant (B = -0.189; SE = 0.116; *P* = 0.102; 95% CI − 0.417, 0.037) (Fig. 3). In addition, meta-regression results to determine the effect of the sample size (*B* = − 0.049; SE = 0.034; *P* = 0.197; 95% CI − 0.011, 0.002) and the duration of follow-up after the intervention (*B* = − 0.025; SE = 0.062; *P* = 0.692; 95% CI − 0.154, 0.103) on the difference of the pooled weighted mean were not statistically significant but showed an indirect association between these variables and the desired effect size (Fig. 3).

Based on the different doses of misoprostol, the weighted mean of the duration of induction was examined in the two groups. The results have been shown in Table 2. The results showed if misoprostol was equally prescribed for the both groups in doses of 50 mg, the weighted mean of the induction duration per hour would be 0.31 h (i.e., approximately 19 min) in mothers who orally took misoprostol compared to ones who vaginally received the drug (WMD: 0.31; 95% CI 0.23, 0.39; I2 44.07%; *P* 0.09). If the dose was 50 mg in the oral misoprostol group and 25 mg in the vaginal misoprostol group, the induction time would increase by 0.48 h (i.e., 29 min) (WMD: 0.48; 95% CI 0.38, 0.58; I2 76.36%; *P* 0.05). In addition, if the drug dose in the both groups was 25 mg, the induction duration would increase by 0.57 h (i.e., 34 min) (WMD 0.57; 95% CI 0.39, 0.75; I2 59.14%; P 0.08) (Table 2).

**Table 2:** Comparison of pooled weighted mean difference of induction labor length in oral and vaginal misoprostol groups based on differ doses

Outcomes	Variables	Effect size (hours)		Heterogeneity assessment			Publication bias		
		WMD	% 95 CI	I^2^ (%)	Q	*P* value	*B*	SE	*P* value
Induction labor length	Oral misoprostol (50 mg)/Vaginal misoprostol (50 mg)	0.31	0.23; 0.39	44.07	21.00	0.09	0.20	0.09	0.29
	Oral misoprostol (25 mg)/Vaginal misoprostol (25 mg)	0.57	0.39; 0.75	59.14	48.90	0.08	0.59	0.10	0.33
	Oral misoprostol (50 mg)/Vaginal misoprostol (25 mg)	0.48	0.38; 0.58	76.36	5.70	0.05	0.33	0.20	0.73

### Other maternal outcomes

**Fig. 4:**
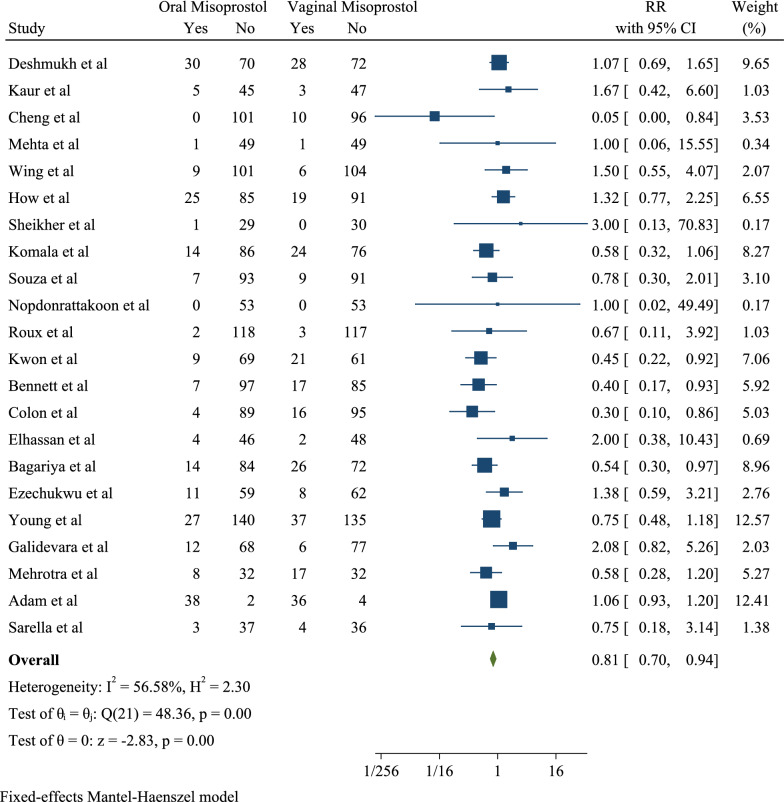
Forest plot for pooled risk ratio of Apgar < 7 at 1 min in oral than vaginal misoprostol groups

Studies were reviewed based on the Apgar score in the first minute, of which 22 had established an association between the Apgar score less than 7 in the first minute and orally and vaginally receiving misoprostol. Among the articles, the highest risk ratio (3.00) was observed in Sheikher et al. study with a confidence interval of 0.13 to 70.83 and the lowest risk ratio (0.05) was seen in Cheng et al. study with a confidence interval of 0.00 to 0.84. After combining the results of these studies, the pooled risk ratio was 0.81 with a confidence interval of 0.70 to 0.94 (I2 56.58% and *P* 0.00). Therefore, the ratio of the Apgar score less than 7 in the first minute can be said to be 0.81 in cases who orally took the drug compared to those who vaginally took misoprostol. Therefore, it was 19% lower in the oral group than the vaginal one (Fig. 4).

**Fig. 5:**
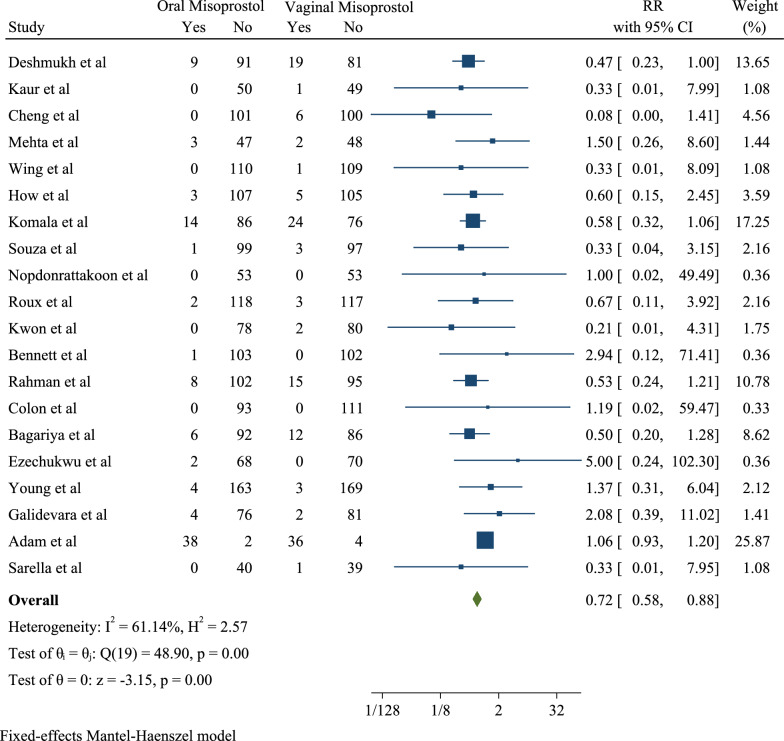
Forest plot for pooled risk ratio of Apgar < 7 at 5 min in oral than vaginal misoprostol groups

Also, 20 studies examined the Apgar score in the fifth minute and determined the association between the Apgar score less than 7 in the fifth (Fig. 5) minute, oral and vaginal misoprostol. Among the articles, the highest risk ratio (5.00) was observed in Ezechukwu et al. study with a confidence interval of 0.24 to 102.30 and the lowest risk ratio (0.08) was seen in Cheng et al. study with a confidence interval of 0.00 to 1.41. After combining the results of these studies, the pooled risk ratio was 0.72 with a confidence interval of 0.58 to 0.88 (I2: 61.14% and P: 0.00). Therefore, the ratio of the Apgar score less than 7 in the fifth minute can be concluded to be 0.72 in cases who orally took the drug compared to those who vaginally took it. Therefore, it was 28% lower in the oral group than the vaginal one (Fig. 4).

### Subgroup results

The results of this study were divided into three groups of Neonatal (The Apgar < 7 in 1 min, The Apgar < 7 in 5 min, IUGR, Oligohydramnios, MSAF, Non-FHR, Neonatal Death and NICU), Maternal (Tachysystole, Hypertonus, Uterine hyperstimulation, Preeclampsia, PROM, Oxytocin Need, Abortion, LSCS and Cesarean) and Adverse effects (Nausea, Vomiting, Headache and Fever) (Table 3).

**Table 3:** Comparison of maternal, neonatal outcomes and adverse effects in two groups

Outcomes	Variables	Effect Size		Heterogeneity Assessment			Publication Bias		
		RR	% 95 CI	I^2^ (%)	Q	*P* value	*B*	SE	*P* value
Neonatal	Apgar < 7 in 1 min	0.81	0.70; 0.94	56.58	48.36	0.00	0.57	0.16	0.04
	Apgar < 7 in 5 min	0.72	0.58; 0.88	61.14	48.90	0.00	0.57	0.14	0.03
	IUGR	1.01	0.71; 1.43	40.36	5.70	0.68	0.29	0.85	0.73
	Oligohydramnios	0.96	0.79; 1.16	47.35	6.79	0.75	− 0.51	0.48	0.29
	MSAF	1.32	1.10; 1.57	54.71	39.74	0.07	0.49	0.50	0.33
	Non-FHR	0.89	0.69; 1.16	28.10	13.97	0.17	− 1.27	0.57	0.02
	NICU	0.96	0.80; 1.16	22.30	21.88	0.19	− 0.05	0.47	0.91
Maternal	Neonatal Death	0.60	0.15; 2.48	0.00	0.21	0.90	− 0.09	1.71	0.95
	Tachysystole	0.71	0.60; 0.85	69.14	45.36	0.00	− 1.38	0.44	0.00
	Hypertonus	1.81	1.00; 3.46	0.00	4.80	0.78	0.31	0.95	0.74
	Uterine hyperstimulation	0.82	0.69; 0.97	52.26	39.80	0.00	− 1.01	0.27	0.00
	Preeclampsia	0.72	0.42; 1.24	42.90	7.00	0.14	− 0.78	1.60	0.62
	PROM	1.44	1.20; 1.72	36.66	5.11	0.41	− 0.46	0.90	0.61
	Oxytocin Need	1.11	1.03; 1.20	85.70	77.04	0.00	0.78	0.30	0.01
	Abortion	0.67	0.19; 2.34	0.00	1.69	0.48	1.65	0.17	0.45
	Cesarean	1.04	0.93; 1.16	63.16	65.33	0.00	− 0.36	0.50	0.47
	LSCS	1.50	1.06; 2.12	0.00	0.70	0.95	0.20	1.56	0.89
Adverse effects	Nausea	1.28	1.00; 1.72	35.52	15.51	0.11	0.68	0.13	0.01
	Vomiting	1.32	1.00; 1.74	13.30	12.69	0.31	0.89	0.55	0.11
	Headache	0.74	0.22; 2.24	0.00	0.94	0.62	0.69	0.32	0.45
	Fever	0.98	0.63; 1.52	0.36	7.03	0.44	1.22	1.12	0.27

The results in the subgroup of neonatal outcomes for the oral and vaginal groups showed the risk ratio for the outcomes of Apgar < 7 in 1 min was 0.81 (*B* = 0.57; SE = 0.16; *P* = 0.04; I2 = 56.58, 95% CI 0.70, 0.94), Apgar < 7 in 5 min 0.72 (*B* = 0.57; SE = 0.14; *P* = 0.03; I2 = 61.14%, 95% CI 0.58, 0.88), IUGR 1.01 (*B* = 0.29; SE = 0.85; *P* = 0.73; I2 = 40.36%, 95% CI 0.71, 1.43), Oligohydramnios 0.96 (*B* = − 0.51; SE = 0.48; *P* = 0.29; I2 = 47.35%, 95% CI  0.79, 1.16), MSAF 1.32 (*B* = 0.49; SE = 0.50; *P* = 0.33; I2 = 54.71%, 95% CI 1.10, 1.57), LSCS 1.50 (*B* = 0.20; SE = 1.56; *P* = 0.89; I2 = 0.00%, 95% CI 1.06, 2.12) and NICU 0.96 (*B* = − 0.05; SE = 0.47; *P* = 0.91; I2 = 22.30%, 95% CI 0.80, 1.16). Therefore, the risk of the outcomes of Apgar < 7 in 1 min, Apgar < 7 in 5 min, Oligohydramnios and NICU can be concluded to be lower in the oral group than the vaginal one and the risk of the outcomes of IUGR, MSAF and LSCS be higher in the oral group compared to the vaginal group (Table 3).

The results in the subgroup of maternal outcomes for the oral and vaginal groups showed the risk ratio for the outcomes of neonatal death was 0.60 (*B* = − 0.09; SE = 1.71; P = 0.95; I2 = 0.00%, 95% CI 0.15, 2.48), tachysystole 0.71 (*B* = − 1.38; SE = 0.44; *P* = 0.00; I2 = 69.14%, 95% CI 0.60, 0.85), hypertonus 1.81 (*B* = 0.31; SE = 0.95; *P* = 0.74; I2 = 0.00%, 95% CI 1.00, 3.46), uterine hyperstimulation 0.82 (*B* =  − 1.01; SE = 0.27; P = 0.00; I2 = 52.26%, 95% CI 0.69, 0.97), preeclampsia 0.72 (*B* =  − 0.78; SE = 1.60; P = 0.62; I2 = 42.90%, 95% CI 0.42, 1.24), PROM 1.44 (*B* = − 1.46; SE = 0.90; *P* = 0.61; I2 = 36.66%, 95% CI 1.20, 1.72), oxytocin need 1.11 (B = 0.78; SE = 0.30; *P* = 0.01; I2 = 85.70%, 95% CI 1.03, 1.20), non-FHR 0.89 (*B* =  − .27; SE = 0.57; *P* = 0.02; I2 = 28.10%, 95% CI 0.69, 1.16), abortion 0.67 (*B* = 1.65; SE = 0.17; *P* = 0.45; I2 = 0.00%, 95% CI 0.19, 2.34) and cesarean fever 1.04 (*B* =–0.36; SE = 0.50; *P* = 0.47; I2 = 63.16%, 95% CI 0.93, 1.16). As a result, the risk of neonatal Death, Tachysystole, Uterine hyperstimulation, preeclampsia, Non-FHR and Abortion was lower in the oral group than the vaginal group and the risk of outcomes of hypertonus, PROM, oxytocin need and cesarean fever was higher in the oral group than the vaginal group (Table 3).

The results in the subgroup of adverse effects for the oral and vaginal groups also showed the risk ratio for the outcomes of nausea was 1.28 (*B* = 0.68; SE = 0.13; *P* = 0.01; I2 = 35.52%, 95% CI 1.00, 1.72), vomiting 1.32 (*B* = 0.89; SE = 0.55; *P* = 0.11; I2 = 13.30%, 95% CI 1.00, 1.74) and headache 0.74 (*B* = 0.69; SE = 0.32; *P* = 0.45; I2 = 0.00%, 95% CI 0.22, 2.24). The outcome of headache can be concluded to be less in the oral group than the vaginal one and the outcomes of nausea and vomiting be higher in the oral group than the vaginal group (Table 3).

### Risk of bias results

The results of qualitative evaluation of articles based on the Cochrane checklist showed the initial selected studies were low risk (Additional file 1).

## Discussion

Prolonged labor is a major cause of maternal mortality and morbidity. Common causes of prolonged labor include inadequate uterine contractions, malpresentation or position of fetus, inadequate pelvic capacity or fetopelvic disproportion. In addition, arrest of labor progress is one of the causes of primary cesarean section, especially in primiparous mothers [19–21]. Therefore, identifying solutions to reduce labor duration can be very important. In this study, the effect of oral and vaginal misoprostol on the labor induction was investigated from different aspects of labor. The labor duration is the most important factor and misoprostol is used for its faster induction [22]. According to the results of this study, vaginal use of misoprostol can induce labor faster and puts childbirth ahead by an average of 24 min (0.4 h), which can significantly reduce the outcomes during labor and postpartum. However, the study results were not the same in other subgroups showed that other factors than how misoprostol was used could affect labor outcomes. In this study, the outcomes were divided into 3 categories of Neonatal, Maternal and Adverse effects. In all three categories, some outcomes were reduced when misoprostol was taken orally and some reduced when misoprostol was taken vaginally. Therefore, to prevent any of these outcomes, making a decision is necessary to be personalized to reduce the risk of the outcome that the person has its risk factor.

In the category of neonatal outcomes, the Apgar scores in the first, and fifth minutes were examined and the results showed the mean of these two indicators was significantly lower in infants whose mothers received misoprostol orally. Based on the results of various published systematic reviews and meta-analyses, especially those published by the Cochrane Center, oral prescription of misoprostol significantly increases the Apgar score than its vaginal administration and in oral use of this drug, the Apgar score less than 7 in the first and fifth minutes is reduced by 19% and 28%, respectively, compared to its vaginal consumption [21, 23–25]. These results are in line with the ones of the present meta-analysis.

The results showed meconium-stained amniotic fluid was 32% higher in infants of mothers who orally took misoprostol than ones whose mothers vaginally received the drug. The rest of the variables examined in the section of neonatal outcomes did not show a significant association.

In the category of maternal outcomes, the rate of premature rupture of membranes in oral consumption was 44% higher than vaginal consumption, and this showed, although the induction duration was longer in the group of mothers taking oral misoprostol than those taking the vaginal drug, rupture of the membranes occurred earlier in this group. On the other hand, in the oral misoprostol group, tachysystole was 29% lower than the vaginal misoprostol group and this finding was different from previous published results. For example, in the study of Galidevara et al. [26], the results showed tachysystole was lower in the vaginal group. The reason for this difference can be attributed to the difference in doses consumed by the study groups or it can even be attributed to the studied populations and the difference in their clinical status and pregnancy. Based on the results of previous studies, tachysystole does not increase neonatal complications [27, 28]. The results of the present meta-analysis showed, although tachysystole at similar doses was higher in vaginal use than oral and the non-reassuring fetal heart rate tracing was higher in vaginal use, the rate of neonatal hospitalization in NICU was not significantly different (4% fewer hospitalizations in NICU in the oral group).

Based on the results of the present meta-analysis, the need for oxytocin in oral administration was 11% higher than vaginal consumption. One of the outcomes of the research was uterine hyperstimulation, the results of the present meta-analysis showed it was 18% lower in mothers taking oral misoprostol than those taking the vaginal drug. These results can be attributed to the neonatal outcome of the non-reassuring fetal heart rate tracing in the present meta-analysis, which was 11% lower in oral administration than vaginal prescription. In previous studies, the results showed increased uterine hyperstimulation could have an increasing effect on non-reassuring fetal heart rate tracing [27–30]. These results were in line with the findings of the meta-analysis, because this study showed in mothers taking oral misoprostol, Uterine hyperstimulation was less and lead to a decrease in non-reassuring fetal heart rate tracing. Regarding the side effects of misoprostol, gastrointestinal side effects were higher in the oral group than the vaginal one, so that nausea and vomiting were 28% and 32% higher in the oral group, respectively. The complication of headache in oral administration was 26% lower than vaginal and for fever, statistical results were not significant. These different effects may be due to the mechanism of action of the two methods of misoprostol consumption, because when orally taken, it shows its effect more systemically, and it may be better able to avoid systemic effects, such as headache. It prevents contraception, and when vaginally taken, its topical effect is stronger and can better and more effectively reduce outcomes, such as PROM [3, 21, 23]. In general, the use of vaginal or oral misoprostol should be decided on a case-by-case basis. The results of the present meta-analysis showed with the use of vaginal misoprostol, uterine contractions were more frequent, and the baby was born earlier, but the Apgar score in the infant and other neonatal outcomes were not appropriate due to lack of or insufficient blood supply to the infant, while the Apgar score and hospitalization in NICU were better in mothers who consumed oral misoprostol. Therefore, it can be concluded that on a case-by-case basis and according to the conditions of the mother and baby, it should be decided to use this drug vaginally or orally. On the other hand, the need for oxytocin in oral misoprostol consumers was higher than vaginal users, because this drug used when the uterine contractions are not enough. The results of the present meta-analysis showed the rate of uterine contractions was lower in oral misoprostol but in contrast the need for oxytocin was higher in this group. There are some hypotheses about premature rupture of membranes and increase in non-reassuring fetal heart rate tracing. The clinical justification is that premature rupture of membranes increases the pressure on the umbilical cord and may lead to increase in non-reassuring fetal heart rate tracing. The results of the present meta-analysis refute this hypothesis. Oral misoprostol users had earlier and more rupture of membranes, which was an interesting result in its own right, but the risk of non-reassuring fetal heart rate tracing was lower in the oral misoprostol group that the vaginal misoprostol group.

The present meta-analysis was performed by analyzing 33 clinical trial studies in which one group of pregnant mothers was given only oral misoprostol and the other group was given only vaginal misoprostol to measure and compare their different pregnancy and neonatal outcomes. The main difference between this meta-analysis and previous ones, especially the Cochrane Center meta-analyses, was that the main study outcome, the duration of labor, was calculated and analyzed as a weighted average, while in previous meta-analyses, this outcome was reported as the risk ratio or odds ratio. On the other hand, the present meta-analysis is the most complete in terms of examining and comparing different maternal and neonatal outcomes. Based on the heterogeneity percentages, all sources of heterogeneity can be inferred to be identified among published clinical trial studies. In this meta-analysis, subgroup analyses were performed based on all possible variables reported in published clinical trial studies.

One of these subgroups analyses was based on different doses of the drug in the two groups and the results showed the labor duration was different for different doses. The main factor in the difference between the results of published clinical trial studies comparing the effect of the labor duration in the two groups of oral and vaginal misoprostol users can be said to be the use of different doses of misoprostol. Therefore, future clinical trial studies should consider this. These studies are necessary in the future, because this drug is currently the most economical medicine in low- and middle-income countries and its use is possible to be continued or even increase with different doses. One of the main limitations of this study was the lack of a sufficient number of studies to compare different outcomes at different doses of misoprostol in the two groups. Therefore, multicenter clinical trial studies with a large sample size and different doses for pregnant mothers are suggested to compare the important maternal and neonatal outcomes.

## Conclusion

The results of the present meta-analysis showed that the oral use of misoprostol was better than vaginal route of administration in several aspects. The labor duration was longer in the oral group, but it had fewer adverse pregnancy and neonatal side effects. In contrast, vaginal use of misoprostol increased uterine contractions. Therefore, it can be inferred that currently, clinical specialists can decide to use this drug orally or vaginally on a case-by-case basis, depending on the condition of the pregnant mother and the baby. However, to provide more accurate evidence and information about the optimal oral or vaginal dose of this drug, multicenter clinical trial studies with a large sample size are required.

## Supplementary Information

**Additional file 1: Figure S1.** Risk of bias graph: review authors' judgements about each risk of bias item presented as percentages across all included studies.

## Data Availability

Data are available and can be accessed from the corresponding author with reasonable inquiry.

## Abbreviations

PRISMA: The Standards of Preferred Reporting Items for Systematic Reviews and Meta-Analyses
NICU: Neonatal Intensive Care Unit
IUGR: Intrauterine Growth Restriction
PROM: Premature Rupture of Membranes
SMD: Standardized Mean Difference
FHR: Fetal Heart Rate
MSAF: Meconium-Stained Amniotic Fluid
LSCS: Lower Segment Caesarean Section

## References

[1] Cunningham FG (2010). Overview of obstetrics. Williams obstetrics.

[2] Ghasemi V (2018). Effective interventions for the induction of labor: a systematic review. Iran J Obstet, Gynecol Infertil.

[3] Takakura S (2021). The successful use of nitroglycerin for uterine hyperstimulation with fetal heart rate abnormality caused by a controlled-release dinoprostone vaginal delivery system (propess): a case report. Medicina.

[4] Tan P (2008). Predictors of newborn admission after labour induction at term: Bishop score, pre-induction ultrasonography and clinical risk factors. Singapore Med J.

[5] Nagpal MB, Raghunandan C, Saili A (2009). Oral misoprostol versus intracervical prostaglandin E2 gel for active management of premature rupture of membranes at term. Int J Gynecol Obstet.

[6] Hatfield AS, Sanchez-Ramos L, Kaunitz AM (2007). Sonographic cervical assessment to predict the success of labor induction: a systematic review with metaanalysis. Am J Obstet Gynecol.

[7] Chanrachakul B, Herabutya Y, Punyavachira P (2002). Randomized trial of isosorbide mononitrate versus misoprostol for cervical ripening at term. Int J Gynecol Obstet.

[8] Lin MG (2005). Misoprostol for labor induction in women with term premature rupture of membranes: a meta-analysis. Obstet Gynecol.

[9] Wu H-L (2017). Misoprostol for medical treatment of missed abortion: a systematic review and network meta-analysis. Sci Rep.

[10] Kunt C (2010). Randomized trial of vaginal prostaglandin E2 versus oxytocin for labor induction in term premature rupture of membranes. Taiwan J Obstet Gynecol.

[11] Malik HZ (2010). Sublingual versus oral misoprostol for induction of labour in prelabour rupture of membranes at term. J Coll Physicians Surg Pak.

[12] Abdelhakim AM, Gadallah A-H, Abbas AM (2019). Efficacy and safety of oral vs vaginal misoprostol for cervical priming before hysteroscopy: a systematic review and meta-analysis. Eur J Obstet Gynecol Reprod Biol.

[13] Chen W (2016). A systematic review and network meta-analysis comparing the use of Foley catheters, misoprostol, and dinoprostone for cervical ripening in the induction of labour. BJOG: Intern J Obstet Gynaecol.

[14] Batukan C (2008). Cervical ripening before operative hysteroscopy in premenopausal women: a randomized, double-blind, placebo-controlled comparison of vaginal and oral misoprostol. Fertil Steril.

[15] Has R (2002). Comparison of 25 and 50 μg vaginally administered misoprostol for preinduction of cervical ripening and labor induction. Gynecol Obstet Invest.

[16] Guha K (2015). Isosorbide mononitrate versus misoprostol for cervical ripening and induction of labour at term. Mymensingh Medical Journal: MMJ.

[17] Haghighi L (2013). Intravaginal isosorbide dinitrate or misoprostol for cervical ripening prior to induction of labour: a randomised controlled trial. J Obstet Gynaecol.

[18] Moher D (2015). Preferred reporting items for systematic review and meta-analysis protocols (PRISMA-P) 2015 statement. Syst Rev.

[19] Ronel D (2012). Trends, risk factors and pregnancy outcome in women with uterine rupture. Arch Gynecol Obstet.

[20] Danielian P (1999). Misoprostol for induction of labour at term: a more effective agent than dinoprostone vaginal gel. BJOG: An International J Obstet Gynaecol.

[21] Shetty A, Danielian P, Templeton A (2001). A comparison of oral and vaginal misoprostol tablets in induction of labour at term. Br J Obstet Gynaecol.

[22] Nassar AH (2007). A randomised comparison of patient satisfaction with vaginal and sublingual misoprostol for induction of labour at term. BJOG An Internat J Obstet Gynaecol.

[23] Cheung K (2022). Clinical algorithms for management of fetal heart rate abnormalities during labour. BJOG: Internat J Obstet Gynaecol.

[24] Hofmeyr G, Gulmezoglu A, Alfirevic Z (1999). Misoprostol for induction of labour: a systematic review. BJOG: Internat J Obstet Gynaecol.

[25] Kerr RS (2021). Low-dose oral misoprostol for induction of labour. Cochrane Database Syst Rev.

[26] Galidevara C, Chaturvedula L, Habeebullah S (2018). Comparison of oral, vaginal and sublingual misoprostol for induction of labour in premature rupture of membranes after 34 weeks of gestation: a randomized controlled trial. Int J Reprod Contracept Obstet Gynecol.

[27] Wing DA (1995). A comparison of misoprostol and prostaglandin E2 gel for preinduction cervical ripening and labor induction. Am J Obstet Gynecol.

[28] Wing DA, Ortiz-Omphroy G, Paul RH (1997). A comparison of intermittent vaginal administration of misoprostol with continuous dinoprostone for cervical ripening and labor induction. Am J Obstet Gynecol.

[29] Wing DA (1995). Misoprostol: an effective agent for cervical ripening and labor induction. Am J Obstet Gynecol.

[30] Verspyck E, Sentilhes L (2008). Abnormal fetal heart rate patterns associated with different labour managements and intrauterine resuscitation techniques. J Gynecol Obstet Biol Reprod.

