# Supplementary material for: Comparison of the effect of oral and vaginal misoprostol on labor induction: updating a systematic review and meta-analysis of interventional studies
Source: Eur J Med Res. 2023 Jan 27;28:51. doi: 10.1186/s40001-023-01007-8 (PMC9881312; doi:10.1186/s40001-023-01007-8)
Supplement: Supplementary file 1 — Additional file 1: Figure S1. Risk of bias graph: review authors' judgements about each risk of bias item presented as percentages across all included studies. [file 40001_2023_1007_MOESM1_ESM.docx]

**
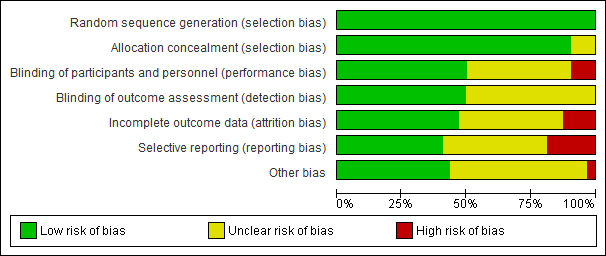
**

**Figure 1: Risk of bias graph: review authors' judgements about each risk of bias item presented as percentages across all included studies.**
